# Radiation doses with various body weights of phantoms in brain 128-slice MDCT examination

**DOI:** 10.1093/jrr/rrz029

**Published:** 2019-06-14

**Authors:** Hung-Chih Lin, Te-Jen Lai, Hsien-Chun Tseng, Ching-Hsiang Wang, Yen-Ling Tseng, Chien-Yi Chen

**Affiliations:** 1 Institute of Medicine, Chung Shan Medical University, Taichung, Taiwan, Republic of China; 2 Department of Radiology, Lukang Christian Hospital of Changhua Christian, Medical Foundation, Lukang, Taiwan, Republic of China; 3 Department of Psychiatry, Chung Shan Medical University Hospital, Chung Shan Medical University, Taichung, Taiwan, Republic of China; 4 School of Medicine, Chung Shan Medical University, Taichung, Taiwan, Republic of China; 5 Department of Radiation Oncology, Chung Shan Medical University Hospital, Chung Shan Medical University, Taichung, Taiwan, Republic of China; 6 Department of Medical Imaging and Radiological Sciences, Chung Shan Medical University, Taichung, Taiwan, Republic of China

**Keywords:** computed tomography, effective dose, thermoluminescent dosimeters, Rando phantom, ICRP 103

## Abstract

The effective dose (H_E_) and organ or tissue equivalent dose (H_T_) for use in brain computed tomography (CT) examinations with various body weights were evaluated. Thermoluminescent dosimeters (TLD-100H) were inserted into Rando and five anthropomorphic phantoms. These phantoms were made of polymethylmethacrylate (PMMA), according to the specifications of ICRU 48, with masses from 10 to 90 kg. Brain CT examinations were conducted, scanning the maxillae from the external auditory meatus to the parietal bone using a 128-slice multi-detector CT (MDCT) scanner. To reduce errors, three independent trials were conducted. Calculated H_E,TLD_, based on the weighting factor recommended by ICRP 103, was 1.72 ± 0.28 mSv, which slightly exceeds the H_E,DLP_ of 1.70 mSv, that was calculated from the dose–length product (DLP) of the Rando phantom. This experiment yielded H_E,TLD_ values of ICRP 103 from the highest 1.85 ± 0.28 (90 kg) to the lowest 1.47 ± 0.22 (10 kg) mSv. H_E,TLD_ (mSv) = 5.45×10^−3^ W(kg) + 1.361, with an *R*^2^ of 0.87667. Using the DLP protocol, H_E,DLP_ was estimated from CTDI_vol_ that was recorded directly from the console display of the CT unit and multiplied by the conversion coefficient (*k*) recommended by the ICRP 103. Finally, the experimental results obtained herein are compared with those in the literature. Physicians should choose and adjust protocols to prevent the exposure of patients to unnecessary radiation, satisfying the as low as reasonably achievable (ALARA) principle. These findings will be valuable to patients, physicians, radiologists and the public.

## INTRODUCTION

According to statistics from the Ministry of Health and Welfare for 2016 [1], the two leading causes of death in Taiwan from 2007 to 2016 were malignant tumor and heart disease. Brain computed tomography (CT) examinations were the most common CT examination, representing 36.8% of all such examinations in Taiwan from 2000 to 2013 [2]. Chen *et al.* stated that the annual CT examination frequency per 1000 population increased by an average of 8.1% per year from 1997 to 2008 [3]. Hu *et al.* found that the annual frequency of CT scans increased from 11.1% in 2009 to 17.7% in 2013, and that the frequency increased for all age groups in Taiwan [4]. The number of CT examinations increased rapidly, with an average annual growth rate of 7.6%, and this trend was similar to those in other countries [5]. Patients undergoing CT examinations range from neonates to oversized adults.

The increasing clinical use of pediatric brain CT scans has raised concerns about their potential detrimental effects on the health of children [6–15]. CT scans are recognized as a higher radiation dose modality than other imaging modalities. Fujii *et al.* and Feng *et al.* presented detailed dose data for pediatric CT examinations [9, 11]. The increasing use of CT has raised particular concerns about the possible detrimental effects of this extra radiation, especially on the health of children. CT examinees always ask physicians and radiologists to tell them how high the out-of-field doses to normal tissue or organs are during an examination. Since the introduction of 128-slice brain CT scanners, short CT data acquisition times and high imaging qualities could be achieved. Since a patient is exposed to significant radiation, the extra doses delivered to adjacent normal organs during brain CT examinations using a 128-slice CT scanner following the manufacturer’s instructions for routine clinical imaging should be evaluated.

The effective dose (H_E,TLD_) and organ or tissue equivalent dose (H_T_) were calculated herein using a thermoluminescent dosimeter (TLD-100H) approach with Rando (Radiology Support Devices, Long Beach, British Columbia, Canada) and five tissue equivalent phantoms, which served as patient substitutes to assess radiation doses during brain CT examinations. In this work, TLDs were inserted into an organ or tissue of each phantom during an axial brain scan. H_T_ values were estimated from TLD measurements that were positioned inside and on the surface of the phantom.

A simple equation can be used to estimate H_E,TLD_ for brain CT examinations of phantoms with various body weights [16, 17]. H_E,DLP_ values were calculated herein using the computed tomography dose index (CTDI_vol_) and the dose–length product (DLP), displayed on the console monitor of the 128-slice CT scanner; these values were then multiplied by the conversion coefficient (*k*) recommended by ICRP 103 [18, 19]. The DLP conversion method is commonly used in clinical practice owing to its simplicity and the ready accessibility of H_E,DLP_.

The experimental results obtained herein are compared with those in the literature. A suitable protocol is strongly recommended to prevent unnecessarily radiating patients and to satisfy the as low as reasonably achievable (ALARA) principle [20–23].

## MATERIALS AND METHODS

### 128-Slice multi-detector computed tomography

At Lukang Christian Hospital (LKCH), brain CT examinations of patients were conducted using 128-slice multi-detector computed tomography (MDCT) (Brilliance; Philips Healthcare, the Netherlands) following the manufacturer’s standard protocol. Extended Brilliance Workspace v4.0 was used to process and analyze the data. The scan conditions were fixed for routine clinical imaging. Before the CT examination began, a ‘surview’ of the phantom was recorded in the CT to plan the examination scan length. Table 1 presents the detailed technical parameters for patients of various body weights during routine brain CT examinations.

**Table 1.** Imaging parameters for six phantoms for routine brain CT examinations^a^

Phantom	Rando	Anthropomorphic				
Weight (kg)	70	10	30	50	70	90
Average tube currents (mA)	400	250	300	350	400	400
Rotation arcs (°)	420	360	360	420	420	420
Rotation time (s)	1.0	0.75	1.0	1.0	1.0	1.0
CTDI_vol_ (mGy)	69.2	42.7	51.1	60.1	69.2	69.2
Scan length (cm)	12.5	6.4	9.2	10.8	12	14.4
DLP (mGy cm)	896.8	302.2	517.8	666.3	896.8	996.5

^*a*^Current tube voltage setting at 120 kVp, collimator 10 mm, beam pitch 1.

### Anthropomorphic phantoms

**Fig. 1.**
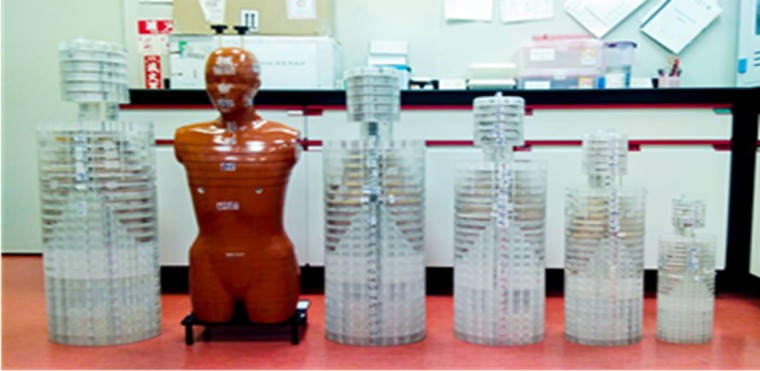
Rando and five anthropomorphic phantoms used as patient substitutes.

Measurements of H_T_ were made using TLDs that were implanted at tissue and organ positions in Rando and polymethylmethacrylate (PMMA) phantoms. It included (i) 400 mAs, (ii) 1.5 s total rotate time, (iii) 10 mm collimation, (iv) 5 mm thickness and (v) 12.5 cm scanned length. The H_E_ of the 70 kg PMMA phantom was verified with that of the Rando phantom. For patients with various body mass, 10–90 kg PMMA phantoms were preset to simulate patients undergoing brain CT examinations. The exposure doses were greatly affected by the patients’ weight, yet the H_E,DLP_ only roughly estimated the given kVp and mAs of the CT intensity with scanned length. Thus, a comprehensive analysis of H_E,TLD_ on the basis of various weights is essential and critical in reality. The standard anthropomorphic phantom, the Rando phantom, represents an adult and has holes for TLDs. The Rando phantom used herein comprised 35 numbered sections that represented the trunk of a man 170 cm tall with a mass of 70 kg [24]. The phantom comprised a human skeleton that was embedded in the anthropomorphic material. The specified densities of the components of the anthropomorphic phantom were 0.98 g cm^−3^ for the soft tissue, 2.70 g cm^−3^ for the skeleton and 0.32 g cm^−3^ for the lungs [25]. Anthropometrically shaped skeletons, constructed from epoxy-resin and PMMA, were used to simulate humans [26]. The PMMA phantoms were based on a general human design. Each had 31 sections, representing the head, neck, torso and abdomen, but without arms or legs. Each phantom was based on the GSF-Forshungszentrum fur Umwelt und Gesundheit (Germany) mathematical models, and the lung masses were based on the ICRP reference man. The densities of the materials were as follows: that of the lung anthropomorphic was 0.296 g cm^−3^; that of the skeleton-cortical-bone anthropomorphic was 1.486 g cm^−3^; and that of the anthropomorphic was 1.105 g cm^−3^. Figure 1 presents the outer appearance of these phantoms.

Table 2 shows the dimensions and physical properties of the phantoms, and the weight and age that correspond to people living in Taiwan [1, 26].

**Table 2.** Dimension and physical properties of Rando, anthropomorphic phantoms and corresponding to age in Taiwan^a^

Phantom	Rando	Anthropomorphic				
Weight (kg)^*b*^	70	10	30	50	70	90
Height (cm)^*b*^	94.5	50	78	84	93	112
Weight (kg)^*c*^	34.5	6.75	19.0	31.5	44.1	57
cm section^−1^	2.5	1.6	2.3	2.7	3.0	3.6
Age (year)	Adult	1	10	15	Adult	Adult

^*a*^available in ref [1].

^*b*^Original design from ref [26].

^*c*^Without arms and legs.

**Fig. 2.**
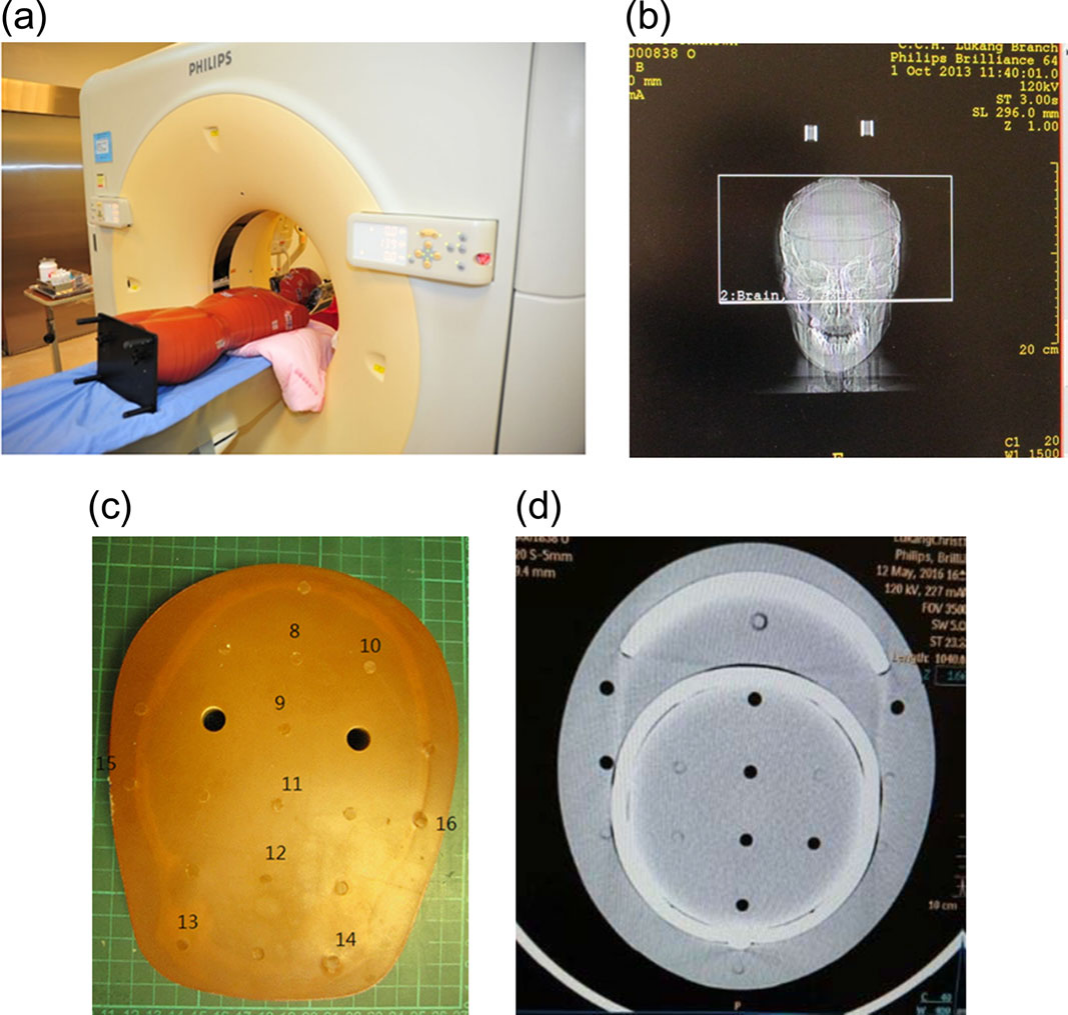
(a) Rando phantom in the 128-slice CT. (b) View of the Rando phantom. (c) Details of the TLDs in the third section, which represented the brain. (d) Medical imaging of the third section of the 30 kg PMMA phantom.

The phantoms were positioned on a patients’ couch and aligned with the isocenter of the gantry using a laser positioning system. Figure 2a presents the Rando phantom in the 128-slice CT of LKCH, Fig. 2b the surview of the Rando phantom, Fig. 2c details of the TLDs in the third section, which represented the brain, and Fig. 2d a medical image of the third section of the 30 kg PMMA phantom.

### Assessment of H_E,TLD_ using the TLD approach

A well-trained senior radiologist (H C Lin) with 10 years experience performed routine brain CT examinations. The examination is whole-brain area axial scans from the maxillae, including the external auditory meatus, to the parietal bones (Fig. 2b).

In 2007, ICRP assigned weighing factors to 14 organs and a group of remainder organs for the purposes of calculating total H_E,TLD_. Only the brain, salivary glands and skin were directly exposed during the brain CT herein. Sensitive organs and tissues were located by visually comparing the phantom sections with anatomical cross-sections from CT examination.

When the absorbed dose in the tissue or organ (D_T_) was measured at several points at a fixed distance from the CT scanning center using TLDs, those values obtained for a particular organ were averaged to obtain a representative dose for that organ (Table 3). The TLDs were also placed at the lens of the eye and at the thyroid to evaluate tissue reactions. Numerous TLDs were placed in each section of each large organ, such as the brain, and the mean estimated absorbed dose was taken as D_brain_. For smaller organs, such as the lens, absorbed doses were obtained by averaging three TLDs in one bag. D_T_ was obtained from these three TLDs that were inserted into the centroid of the organ. Muscles were excluded from the remainder since they extended throughout the body, making H_muscles_ difficult to measure.

**Table 3.** Weighting factor (W_T_) of organ or tissue recommended by ICRP 103 as well as number of TLD-100Hs inserted into the phantom

Organ/Tissue	Measured points	W_T_	Number of TLDs
Breast	Breast	0.12	3
Bone marrow		0.12	
	C-spine	0.06	6
	Thighbone femur	0.06	3
Colon	Colon	0.12	3
Lung	Lung	0.12	3
Stomach	Stomach	0.12	3
Gonads	Gonads	0.08	3
Bladder	Bladder	0.04	3
Esophagus	Esophagus	0.04	3
Liver	Liver	0.04	3
Thyroid	Thyroid	0.04	6
Bone surface		0.01	3
Brain	Brain	0.01	39
Salivary gland	Salivary gland	0.01	6
Skin	Skin	0.01	93
Remainder		0.12	
	Heart	0.03	3
	Pancreas	0.03	3
	Kidney	0.03	3
	Small intestine	0.03	3
Lens			6
Total		1.000	207

The calculation of H_E,TLD_ values for a large number of organs and tissues is recommended by ICRP 103. To determine the organ- or tissue-equivalent dose, H_T_, the following equation is used.

(1) $$H_{T}{=D}_{T}{\times W}_{R}$$

where W_R_ is the radiation weighting factor (W_R_ = 1 Sv/Gy for X-rays).

To calculate the effective dose, H_E,TLD_, the products of H_T_ and W_T_ are summed.

(2) $$H_{E,\mathrm{TLD}}{=\Sigma }_{T}H_{T}{\times W}_{T}$$

Some organs and tissues are explicitly listed in Table 3; others are classified as ‘remainder tissues’. To make dosimetry evaluations for the remainder tissues, organs/tissues which were within or close to the radiation field were selected. For these tissues, H_E,TLD_ was calculated from the product of W_T_ for the remainder tissues and H_T_, divided by the total number of remainder organs. The W_T_ values have been demonstrated to be broadly applicable to both adults and children, although the best method for evaluating the risk associated with a brain CT examination requires knowledge of H_T_ and age-specific organ risk factors [18].

The locations of the organs from the head to the gonads, including the remainder organs, were used in evaluating H_T_. A total of 207 TLDs were used. To minimize the errors coming from scattering and absorption in/at TLDs, three TLDs were placed in each bag to obtain D_T_ at a particular location [16, 24]. For brain CT imaging, *in vivo* measurements were made inserting TLDs into the brain, while other TLDs were exposed to extra scattered radiation. Fifty-one TLDs were located in the head of the patients. Nine TLDs were used to measure the background radiation in a low background laboratory. The total errors were effectively suppressed by performing three independent trials that involved 31 bags of TLDs that were attached to the surface of the phantom. Table 3 shows the attachment points on organs or tissues that are recommended by ICRP 103, along with the corresponding values of W_T_ [18].

### Accuracy and calibration of TLDs

To calibrate the photon dose and the linearity of the TLDs as well as to reduce experimental errors, the TLDs were pre-calibrated using X-ray beams to those used in the 120 kV, 30 mA on the 128-slice Brilliance CT at LKCH. TLDs were irradiated at doses of 0.5–15 mGy, which includes the prescribed daily fraction dose, at a depth of 5 cm in solid water (CIRS, Norfolk, VA, USA). Solid water was used rather than water to make experimental uncertainty negligible. A Farmer-type ionization chamber of type NE 2571 (Nuclear Enterprises, UK) with a volume of 0.6 ml was positioned in the solid water. The TLD-100H was used owing to its small dimensions and the lower dependence of its response on photon energy, dose rate and the direction of incidence of radiation. Radiation doses were read out from a glow curve of a Harshaw 3500 analyzer (Harshaw, Cleveland, OH, USA) after 24 h of exposure. TLDs were preheated to 70°C at a rate of 10°C s^–1^ by applying 780 V and further heated to a maximum temperature of 300°C. Moreover, after responding adequately to the annealing process, the TLDs could be recycled.

### Evaluation of H_E,DLP_ from measured CTDI_vol_ values

To estimate H_E_,_DLP_, the DLP protocol was used. The CTDI_vol_ value at 120 kV was obtained from dosimetry data during the routine quality assurance program at LKCH and is presented in Table 1. CTDI_vol_ was particularly marked for the brain CT, as the most radiosensitive organ, and the CTDI_vol_ value was increasingly far from the X-ray beam as the size of the phantom increased. The DLP was obtained from measurements that were made on the specific dosimetry phantom with an adult head diameter of 16 cm and an adult body diameter of 32 cm [27]. Some values of H_E,DLP_ for variously sized patients or phantoms have been published [12]. H_E,DLP_ is an indicator of effective dose, but takes no account of the variations in organ sensitivities.

The CTDI_vol_ value that was directly recorded on the console display of the 128-slice CT during the scan was multiplied by the scan length to yield the DLP.

(3) $$\mathrm{DLP}\left(\mathrm{mGycm}\right){=\mathrm{CTDI}}_{\mathrm{vol}}\left(\mathrm{mGy}\right)\times \mathrm{scanned length}\left(\mathrm{cm}\right)$$

The DLP denotes the total energy absorbed during (and therefore the potential biological effect attributable to) the complete scan [18, 19].

(4) $$H_{E,\mathrm{DLP}}\left(\mathrm{mSv}\right)≒k\times \mathrm{DLP}$$

where *k* (mSv mGy^−1^ cm^−1^) is the conversion factor of H_E,DLP_/DLP for the CT scan.

ICRP 103 states that, using this methodology, H_E,DLP_ can be estimated from DLP, which is obtained by most CT systems; *k* values for the Rando and various body weight phantoms are Rando, 0.0019; 10 kg, 0.0053; 30 kg, 0.0027; 50 kg, 0.0019; 70 kg, 0.0019; and 90 kg, 0.0019 mSv mGy^−1^ cm^−1^ [18, 19, 28]. In addition, the protocol of H_E,DLP_ calculation is most commonly used in a clinical setting.

## RESULTS AND DISCUSSION

Brain CT examinations irradiate multiple organs or tissues with various radiation sensitivities. H_E_ values take into account how much radiation is received by an individual tissue as well as the tissue’s relative sensitivity to radiation.

In this investigation, H_E,TLD_ values for brain CT examinations were calculated using the TLD-100H approach. However, radiation doses in brain CTs vary widely with the type of CT scanner and the scan parameters that are set by the medical facility [6]. The H_E,DLP_ protocol was conversion factor (*k*) multiplied the total energy absorbed during the complete brain CT scan. DLP can be calculated from the CTDI_vol_ value, directly recorded on the console display of the CT unit after the imaging of patients, multiplied by the scan length. It is simply and easily obtained by most CT systems from a clinical viewpoint. In contrast, equivalent doses of organs or tissues (D_T_) are essential to measure the specific exposure doses. Thus, in reality, the H_E,TLD_ protocol is the most suitable technique. The H_E,TLD_ protocol is better than H_E,DLP_ and is not used in clinical investigations.

### TLD calibration and uncertainty

The TLD-100H responded linearly to radiation doses from 0.5 to 15 mSv. The conversion factor for the TLD-100H, obtained using the EXCEL linear regression function, was Y(mSv) = −0.768 + 9.978 × TLD (nC), and the square of the correlation coefficient (*R*^2^) was 0.9877 [25].

The precision and accuracy of the dose estimations using TLD-100H are specified by several parameters. The total errors in this study were obtained mostly from (i) TLD-100H counting statistical errors (Δ_counting_) from 3% for measurements within or close to the direct beam to over 10% for measurements well outside the beam, where the measurements are close to the background; (ii) the systematic uncertainties of the 3500 reader (Δ_reader_) have been demonstrated to be <10% (ranging from 5% to 8%); (iii) the uncertainty in W_T_ (Δ_WT_) was set to 5% because the W_T_ was normalized [18]; (iv) the linear calibration (Δ_calibration_) of the X-ray of 128-slice CT was from 3% to 10%; (v) the uncertainty of the locations (Δ_location_) of the TLDs in an organ was 5%; (vi) the uncertainty that arose from non-tissue equivalence effects (Δ_non-tissue_) for the anthropomorphic phantom was set to 5%, because PMMA phantoms are entirely based on ICRU 48 [26]; and (vii) the variations in power fluctuations (Δ_128-MDCT_) coming from 128-MDCT were <2% during monthly clinical quality assurance at LKCH. The total uncertainty (Δ_total_) was derived as the square root of the sum of squares of the individual errors from (i) to (vii). Symbolically, this can be represented as equation 5:

(5) $$\Delta_{total}=\sqrt{{\Delta_{counting}}^{2}+{\Delta_{reader}}^{2}+\Delta {w_{T}}^{2}+{\Delta_{calibration}}^{2}+{\Delta_{location}}^{2}+{\Delta_{non-tissue}}^{2}+{\Delta_{128-MDCT}}^{2}}$$

The total uncertainties ranged from 11.0% to 18.5%. Other phenomena affecting the total uncertainties such as uncertainty in the DLP arose mainly from the calibration of the ion chamber that was used to measure the weighted CTDI (CTDI_W_) and was calculated to be <2%.

### Equivalent doses delivered to organs or tissues (H_T_) of examinees with various body weights

**Fig. 3.**
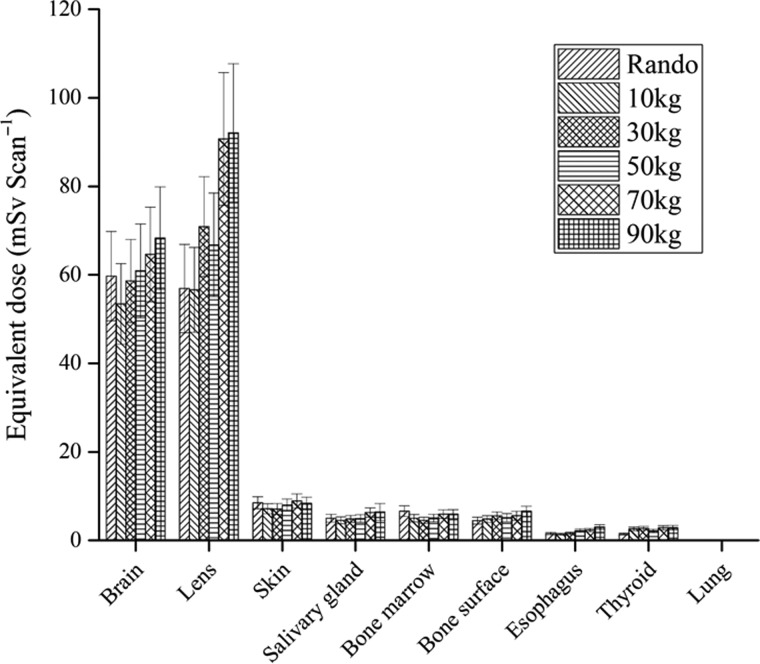
Equivalent doses (mSv) delivered to critical organs in six phantoms during brain CT examination. H_T_ was measured by placing various TLDs in each organ/tissue. Average values and spread over TLDs are shown (bars).

Figure 3 plots the measured H_T_ values that were delivered to the phantom. High H_T_ values were recorded in the brain, lens, skin, salivary gland, bone marrow and bone surface. Parts of the skin and salivary gland were scanned. All these organs had relatively low tissue weighting factors (W_T_) in Table 3. Doses delivered to the red bone marrow (RBM) and bone surface were evaluated from the doses that were measured in various bone tissues. H_E,TLD_ values were calculated as recommended by ICRP 103 [18].

### Entrance surface air kerma or skin dose

**Fig. 4.**
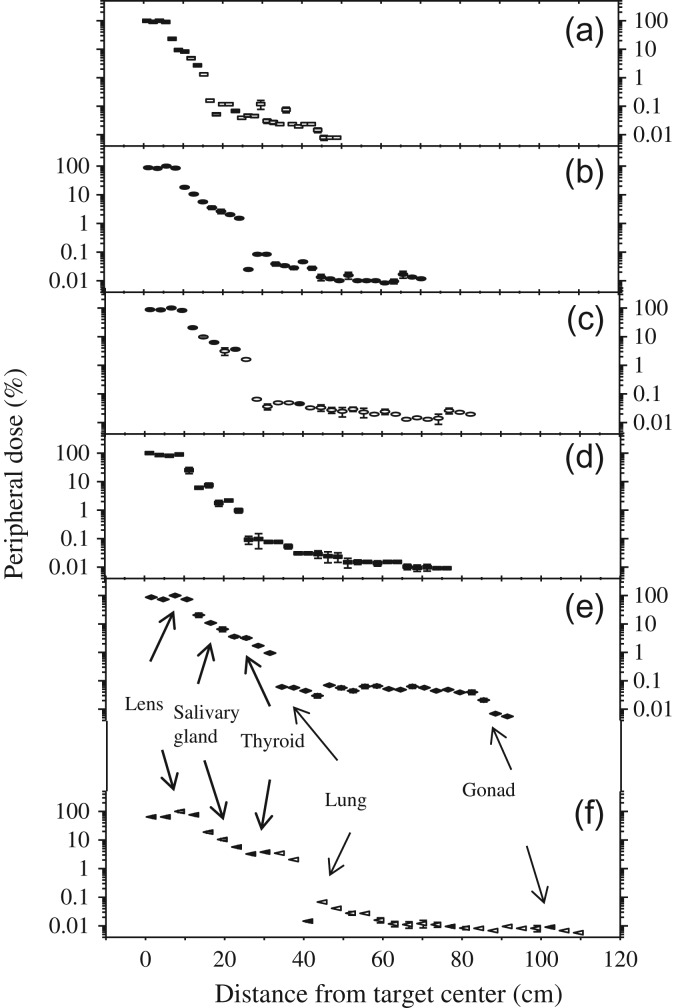
ESAK (%) vs lateral distance (cm) from the CT target center during brain CT examination. (a) 10 kg. (b) 30 kg. (c) 50 kg. (d) Rando. (e) 70 kg. (f) 90 kg phantoms.

Based on 31 measurements (Table 3), estimates reflected the entrance surface air kerma (ESAK) or skin dose at any point in phantoms with various body weights. ESAK at the entrance and the exit of the CT scanner were measured using 93 TLDs that were attached to the surface of each phantom. Most X-rays originated in the target of the 128-slice CT and scanned the first to fifth sections of the PMMA phantom. Figure 4a–f plots ESAK values as a function of measurements over three trials, based on the distance from the scanning edge of the phantom. ESAK at the periphery of the scan volume can have significant variations because of extra irradiation.

ESAK values ranged from 7.13 ± 1.14 mSv (10 kg) to 8.89 ± 1.60 mSv (70 kg). They were normalized independently to 100% of the background CT field for each phantom. Out-of-field doses revealed a gradual reduction in ESAK with increasing distance from the scanning edge in brain CT, as expected. The ESAK values outside the CT volume varied significantly, and decreased as the distance from the CT field increased. ESAK values depended on whether the skin was in or out of the direct beam.

The highest average equivalent doses are the brain, H_brain_, in the brain CT examinations. The H_brain_ value of the Rando phantom, 59.7 ± 10.1 mSv, was ~0.92 times lower than that of the 70 kg phantom, which was 64.6 ± 10.7 mSv. The error bars represent uncertainties in the H_brain_ values. The difference between the Rando and 70 kg phantoms was 7.59%. The large deviation was caused mainly by the skeleton-cortical-bone anthropomorphic density (1.486 g cm^−3^) in the head of the PMMA phantom [24].The density effect is related to the soft tissues, rib or spine inside the PMMA phantoms. The H_brain_ values are much lower than the threshold (~500–2000 mSv) dose for cataractogenesis [18].

Despite angling the gantry to reduce the dose, the lens doses (H_lens_) received by the eyes of the six phantoms during brain CT ranged from 56.6 ± 9.6 mSv (10 kg) to 92.1 ± 15.6 mSv (90 kg). These values are higher than those of Mettler *et al.* of 30–50 mGy [29], but they were still well below the threshold of ~1.5 Gy for cataractogenesis [18].

The thyroid is located in section 10 of the PMMA phantom. It is a radiosensitive organ and, being located on the border edge of the CT scan section, received the highest, 2.86 ± 0.49 mSv (90 kg), to the lowest, 1.42 ± 0.24 mSv (Rando), doses of any organ in the six phantoms. Lungs, breast, colon and gonad thus received relatively low H_T_ in this examination. No significant differences were observed between the estimated H_heart_ and H_gonad_. From the brain CT examinations, organs or tissues that did not lie close to the vicinity of the CT field received approximately the background radiation.

### Effective doses (H_E,TLD_)

**Fig. 5.**
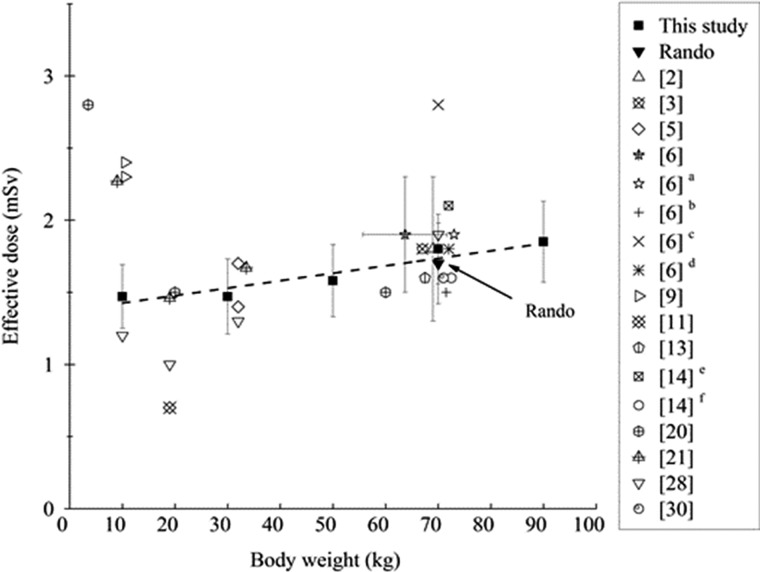
Estimate of H_E,TLD_ as a regression function of various body weights of the phantom compared with others, the coefficient of which was calculated to be *R*^2^ = 0.87667. Error bars represent counting errors.

Figure 5 indicates that the estimated effective doses (H_E,TLD_) increased with body weight. In addition, the H_E,TLD_ that was evaluated accoding to ICRP 103 varied from the highest value of 1.85 ± 0.28 mSv (90 kg) to the lowest value of 1.47 ± 0.22 mSv (10 kg). The regression equation between H_E,TLD_ and the body weight of the examinee, H_E,TLD_ (mSv) = 5.45×10^–3^ W (kg)+1.361, has an *R*^2^ of 0.87667. Because the *R*^2^ in linear regression is high, it reveals that TLD is a good approach for evaluating the H_E,TLD_ of PMMA phantoms during brain CT examinations [25, 30]. The H_E,TLD_ value of the Rando phantom was 1.72 ± 0.28 mSv, which was lower than that of the 70 kg phantom, 1.80 ± 0.24 mSv. H_E,TLD_ values in this study were obtained mostly from the doses of RBM owing primarily to the fact that it had the largest tissue W_T_ of 0.12.

### Effective dose (H_E,DLP_) estimated by ICRP 103

CTDI_vol_ and DLP have been proposed as the most effective doses for establishing diagnostic reference levels. In the DLP protocol, one of the main reasons for the discrepancies is that the relative radioactive sensitivity of an anatomical region does not vary with patient size, as recommended by ICRP 103 [18]. H_E,DLP_ was estimated from CTDI_vol_ that was recorded directly from the console display of the CTDI_vol_ of the 128-slice CT scanner during the quality assurance program at LKCH. H_E,DLP_ values were multiplied by specific normalized conversion factors (*k*), which were obtained from ICRP 103 [12, 18]. Those considerable differences are mainly attributable to differences between the designs of Rando and PMMA phantoms that are used to generate the DLP conversion factors. This indicates that the values of H_E,TLD_, and H_E,DLP_ for the Rando phantom that were calculated using TLD and DLP protocols were 1.72 ± 0.28 mSv and 1.70 mSv, respectively. The slight discrepancy can be explained by the difference between (i) the densities of the Rando and 70 kg PMMA phantoms and (ii) the scanning ranges used. However, the literature from 1998 to 2017 includes no exact scanning ranges [5, 9, 11, 14].

### Limitations

This investigation has many limitations, including the following: (i) inherent variation among TLD-100H dosimeters, TLD chip positioning and directionality error; (ii) a lack of published data on conversion factors (*k*) of the new generation of CT scanners (128-slice CT scanners) for adult and pediatric use; (iii) lack of verification of results in clinical practice as TLDs cannot be used in patients for ethical reasons; (iv) limited evaluation of image noise in the quantitative image quality assessment; (v) effect of scanning range on H_E,DLP_ values as a result of allowing a wider beam width in scanning acquisitions [6]; and (vi) inherent uncertainties in the definition of H_E,TLD_ that arise from the use of an approximate W_T_, averaged over a population. Despite their limitations, TLDs remain the most practically useful devices for obtaining D_T_ and H_T_ values for medical procedures [25].

### Comparison of results with those in other studies

H_E,TLD_ and H_E,DLP_ measurements of phantoms and patients who are undergoing brain and head CT examinations fall in a rather wide range, H_E_ from 0.7 to 2.8 mSv. Fujii *et al.* stated that a comparison of results must consider the particular phantoms and scanning ranges that are used [9]. Overall, the H_E,TLD_ and H_E,DLP_ values in this investigation agree closely with those in Fig. 5 and Table 4, except for the value of 2.8 mSv for a 3.5 kg newborn phantom and 0.7 mSv for a 5-year-old 19 kg phantom [11, 20].

**Table 4.** Comparisons made for the H_E,TLD_ and H_E,DLP_ per scan among Germany, Greece, Italy, Malaysia, Taiwan and the UK

CT	H_E,TLD_ (mSv)	H_E,DLP_ (mSv)	Weight (kg)	Age (years)	Method, phantom, CT examination	Reference
Philips 128	1.72 ± 0.28	1.70	70	Adult	TLD, Rando, brain	This study
Philips 128	1.47 ± 0.22	1.60	10	1	TLD, PMMA phantom, brain	This study
Philips 128	1.47 ± 0.26	1.40	30	9	TLD, PMMA phantom, brain	This study
Philips 128	1.58 ± 0.25	1.27	50	13	TLD, PMMA phantom, brain	This study
Philips 128	1.80 ± 0.24	1.70	70	Adult	TLD, PMMA phantom, brain	This study
Philips 128	1.85 ± 0.28	1.84	90	Adult	TLD, PMMA phantom, brain	This study
4 hospitals		1.9 ± 0.4	55.7–71.7	Adult	Patients, brain, Malaysia, 2016	6
Hospital		1.9		Adult	Patients, brain, Malaysia, 2009	6
Hospital		1.5		Adult	Patients, brain, UK, 2003	6
Hospital		2.8		Adult	Patients, brain, Germany, 2002	6
Hospital		1.8			Patients, brain, Germany, 1998	6
30 hospitals		1.80 ± 0.5		Adult	1754 Patients, head, Taiwan, 2016	2
Sensation 16		1.2	10	1	DLP, ATOM phantom, head	28
Sensation 16		1.0	19	5	DLP, ATOM phantom, head	28
Sensation 16		1.3	32	10	DLP, ATOM phantom, head	28
Sensation 16		1.9	70	Adult	DLP, Rando, head	28
Toshiba 64		2.8	3.5	0	DLP, CIRS newborns phantom, head	20
Toshiba 64		1.5	20	6	DLP, CIRS phantom, head	20
Toshiba 64		1.5	60	Adult	DLP, Japanese male phantom, head	20
Siemens 16	1.4	1.7	32	10	TLD, CIRS phantom, brain	5
Aquilion 64		2.4	10	1	DLP, CIRS infant phantom, head	9
Discovery 750		2.3	10	1	DLP, CIRS infant phantom, head	9
16-slice CT		1.6	63–78	Adult	167 Patients, head, Sudan	30
GE 64		0.7	19	5	DLP, CIRS phantom, head	11
CT in Taiwan		1.8		All age	Patients, head, Taiwan, 2008	3
CT in Taiwan		1.6			146 Patients, head, Taiwan, 2007	13
14 CT scanners		2.1 0.7–3.7	70	Adult	Patients, head, Greece, 2003	14
32 CT scanners		1.6	70	Adult	Patients, head, Italy, 2003	14
Siemen Somoton		2.27	9.36	1	TLD, Cristy phantom, head	21
Siemen Somoton		1.46	19.1	5	TLD, Cristy phantom, head	21
Siemen Somoton		1.67	32.1	10	TLD, Cristy phantom, head	21

Here, H_E,DLP_ is derived from the DLP conversion method and H_E,TLD_ is derived from the TLD approach.

Karim *et al.* reported on 376 brain CT examinations that were performed in many hospitals in Malaysia. The H_E,DLP_ values for patients with weights from 55.7 to 71.7 kg ranged from 1.6 ± 0.7 to 2.1 ± 0.6 mSv [6].

Yeh *et al.* collected brain CT data from 4467 patients in Taiwan from 2000 to 2013, and obtained an H_E,DLP_ value of 1.8 ± 0.5 mSv for head CT examinations (39.8% of 1754 patients) [2]. Chen *et al.* presented estimates of H_E,DLP_ for head CT examinations with different medical modalities in 2008. The averaged H_E,DLP_ value during 3.60×10^5^ head CT examinations was 1.8 mSv [3]. The published H_E,DLP_ values for adults in Taiwan in 2008 and 2007 are 1.8 and 1.6 mSv, respectively, and 1.80 ± 0.5 mSv for adults in Germany in 1998. The Rando phantom dose that was evaluated in this investigation was similar to those reported in Taiwan in 2016, 2008 and 2007, and in Germany in 1998 [2, 3, 6].

Sugimoto *et al.* stated that detailed evaluations of H_T_ and H_E,DLP_ for patients from infants to adults who are undergoing head CT examinations are very important in assessing their risk of cancer. They obtained H_E,DLP_ values for newborn (3.5 kg) and 6-year-old children (20 kg) and for a standard Japanese adult phantom (60 kg) under a non-helical head CT examination using the planner silicon pin-photodiodes approach; the values were 2.8, 1.5 and 1.5 mSv, respectively [20]. The H_E,DLP_ of the newborn phantom, 2.8 mSv, is 1.79 times higher than that obtained from our regression curve in Fig. 5.

Brady obtained an H_E,TLD_ value for a brain CT examination of a 32 kg phantom of 1.4 mSv using the TLD approach and an H_E,DLP_ value of 1.7 mSv using the DLP protocol recommended by ICRP 103. These results were generally lower than those obtained herein [5]. Fujii *et al.* reported that the H_E,DLP_ for a head CT examination of a 1-year-old infant (10 kg) was 2.4 mSv and the H_brain_ values ranged from 28 to 32 mSv [9].

A better agreement of ~10% was found between the H_E,DLP_ that was obtained using the DLP conversion protocol and the H_E,TLD_ that was obtained using the TLD approach, for a Rando during brain CT, as depicted in Fig. 5. The values of H_E,TLD_ herein are close to published data, but higher than 1.6 mSv obtained for 167 adult patients in Sudan with body weights from 63 to 78 kg. An H_E_ value of 2.1 mSv for routine head CT examinations has been obtained in Tanzania [30].

Feng *et al.* used the TLD approach to obtain H_E,DLP_ from brain CT examinations of a 19 kg phantom (5-year-old child). The H_E,TLD_ was 0.7 mSv, which is much lower than that obtained from the regression curve in our study, as depicted in Fig. 5 [11]. Papadimitriou *et al.* evaluated H_E,DLP_ for brain CT examinations of children of various ages using *k* values. Mean H_E,DLP_ values for the Rando phantom of 2.1 mSv in Greece and 1.6 mSv in Italy have been obtained as recommended by ICRP 103 [14]. H_E,TLD_ and H_T_ values for children of various ages under each CT protocol were compared, as reported in a previous study. Chapple *et al.* reported the H_E,DLP_ mean doses in head CT examinations for 1 year olds (9.36 kg) and 5 year olds (19.1 kg). Cristy mathematical phantoms were 2.27 mSv and 1.46 mSv, respectively. Chapple *et al.* demonstrated that during brain CT examination of any particular type, H_E,DLP_ varies greatly with the scan length [21].

### CONCLUSION

This study is the first to measure the H_E,TLD_ and H_E,DLP_ values of patients with various body weights during brain 128-slice MDCT examinations. The variations of CT is <2%. Brain CT examinations were conducted, scanning the maxillae from the external auditory meatus to the parietal bone. The equivalent doses to the brain, lens and skin were extremely high and differed significantly from those to other organs. The H_E_ varied with the type of CT scanner and the scan parameters that were used at the medical facility. These results indicate that the TLD-100H approach is highly sensitive. Effective doses that were calculated using the TLD approach ranged from 1.47 ± 0.22 mSv to 1.85 ± 0.28 mSv. These can be compared with the natural background radiation of >2 mSv. The quantitative dose information herein indicates the relationship between brain CT examinations and radiation dose, and provides practical guidance for optimizing clinical practice in conducting brain CT examinations. Future work should continue to improve brain CT imaging using sensitivity analysis, Taguchi analysis (method) with an indigenous line-paired flat phantom.
